# A Simple Small Size and Low Cost Sensor Based on Surface Plasmon Resonance for Selective Detection of Fe(III)

**DOI:** 10.3390/s140304657

**Published:** 2014-03-07

**Authors:** Nunzio Cennamo, Giancarla Alberti, Maria Pesavento, Girolamo D'Agostino, Federico Quattrini, Raffaela Biesuz, Luigi Zeni

**Affiliations:** 1 Department of Industrial and Information Engineering, Second University of Naples, Via Roma 29, Aversa 81031, Italy; E-Mail: luigi.zeni@unina2.it; 2 Department of Chemistry, University of Pavia, Via Taramelli 12, Pavia 27100, Italy; E-Mails: galberti@unipv.it (G.A.); maria.pesavento@unipv.it (M.P.); girolamo@unipv.it (G.D.); federico.quattrini01@universitadipavia.it (F.Q.); raffaela.biesuz@unipv.it (R.B.)

**Keywords:** plastic optical fiber, surface plasmon resonance, deferoxamine, self-assembled monolayer, iron(III)

## Abstract

A simple, small size, and low cost sensor based on a Deferoxamine Self Assembled Monolayer (DFO-SAM) and Surface Plasmon Resonance (SPR) transduction, in connection with a Plastic Optical Fiber (POF), has been developed for the selective detection of Fe(III). DFO-SAM sensors based on appropriate electrochemical techniques can be frequently found in the scientific literature. In this work, we present the first example of a DFO-SAM sensor based on SPR in an optical fiber. The SPR sensing platform was realized by removing the cladding of a plastic optical fiber along half the circumference, spin coating a buffer of Microposit S1813 photoresist on the exposed core, and finally sputtering a thin gold film. The hydroxamate siderophore deferoxamine (DFO), having high binding affinity for Fe(III), is then used in its immobilized form, as self-assembled monolayer on the gold layer surface of the POF sensor. The results showed that the DFO-SAM-POF-sensor was able to sense the formation of the Fe(III)/DFO complex in the range of concentrations between 1 μm and 50 μm with a linearity range from 0 to 30 μm of Fe(III). The selectivity of the sensor was also proved by interference tests.

## 1. Introduction

Iron is an important metal ion since it is essential in many metabolic pathways. However, the concentration of Fe(III) in biological systems has to be efficiently balanced as both its deficiency and excess can cause various biological disorders [1]. The same holds for environmental systems, such as fresh and seawaters, in which the iron concentration is claimed to be of crucial relevance [2]. Thus new methods for iron analysis are widely required, in particular for *in situ* applications, *i.e.*, not only sensitive, but also robust, rapid, easy to perform and possibly at low cost. Sensor technology is particularly suited to meeting these aims.

Deferoxamine B (DFO), a bacterial hydroxamate siderophore, has a high affinity for Fe(III), so that DFO is one of the most employed drugs used in chelation therapy to remove excess iron from blood and tissues [3,4]. In particular, DFO forms 1:1 Fe-DFO octahedral complex involving the six oxygen atoms of the hydroxamate groups [5]. The terminal amino group is not involved in iron complexation, therefore it is available for anchoring DFO onto a solid phase. On the basis of this strategy, we have previously developed and reported a sensor for Fe(III) based on spectrophotometric transduction called DFO Self-Assembled Monolayers on Mesoporous Silica (DFO SAMMS) [6].

**Figure 1.**
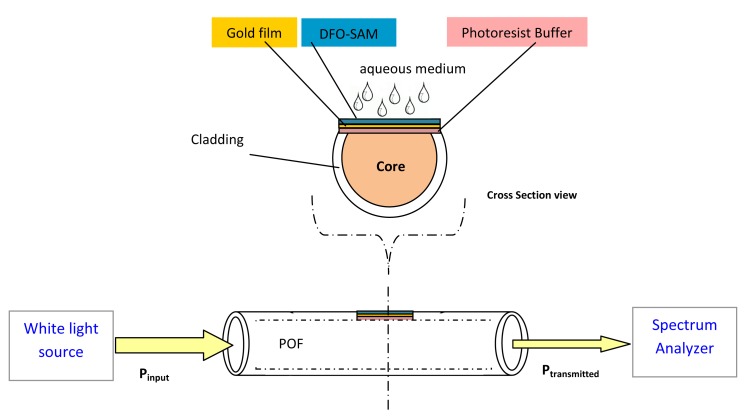
POF chemical sensor based on SPR and experimental setup.

In this work, we present a sensor for Fe(III) with optical transduction based on the surface plasmon resonance phenomenon excited at the interface of a dielectric layer and a gold film, exploiting a multimode Plastic Optical Fiber (POF) as the light guiding structure (see Figure 1). DFO has been anchored to the gold surface as a self-assembled monolayer (SAM) affording a DFO-SAM-modified interface [7]. This configuration should make possible the development of a new, simple and low cost sensor for Fe(III) taking advantage of the complexation of Fe(III) with DFO.

SPR is a very sensitive technique for determining small refractive index changes at the interface between a metallic layer and a dielectric medium. If the surface of the metal is functionalized with a receptor, the combination of the analyte with the receptor can induce a variation of the refractive index at the interface which can be measured, even if it is quite small. For these reasons SPR is widely used as a detection system for sensors operating in different areas of biology and chemistry, as reported in several recent reviews [8–10]. Anchoring the receptor at the gold surface as an SAM is particularly suited to SPR transduction, since the receptor-substrate combination takes place exactly at the gold/external dielectric medium interface.

Most often SPR bio-chemical sensor systems are based on a high refractive index prism coated with a thin metallic layer (Kretschmann configuration). The incidence angle of the light for which the plasmon resonance is induced depends on the refractive index of the dielectric medium. It can be changed over a wide range, and consequently the excitation of the surface plasma waves (plasmons) may exist whatever the surrounding medium, *i.e.*, a gas or a liquid [8]. These sensors are usually bulky, and not easy to miniaturize and require expensive optical equipment. In principle, these aims can be successfully achieved by using waveguide coupling. An optical fiber makes it possible to reduce the sensor cost and dimensions, with the possibility to integrate the SPR sensing platform with miniaturized optoelectronic devices, eventually leading to a “lab-on-a-chip”.

Actually, several configurations based on SPR in silica optical fibers for measuring the refractive index of aqueous media have been described in the recent literature [10,11].

Furthermore, investigations have also been devoted to Plastic Optical Fibers (POF) as they represent an easier to handle platform with mechanical properties making them more resilient, cheaper and safer for *in vivo* determinations.

We recently developed a new geometry for a low cost POF sensor system [12,13] suitable for bio-applications [14] and also for low molecular weight substances determination [15,16].

In the present work, the configuration based on POF is combined with DFO-SAM as the recognition element for Fe(III). Although SPR cannot compete with the detection limit of other analytical techniques for metal ion determination such as ICP-MS, the attractiveness of using SPR sensing lies in the possibility of obtaining data in real time and without labelling either the ligand or the analyte. Thus SPR could be an alternative sensing platform for heavy metal ions.

Different ligands have been proposed for SPR sensing of several metal ions. For example, Mirkhalaf *et al.* described the application of SPR to Cu^2+^, and Cd^2+^ determination using a sensing layer containing dithizone [17]; squarylium dye containing polymeric thin film was used by Ock *et al.* for Cu^2+^ sensing [18], and the detection of Cu^2+^ and Ni^2+^ was achieved by coating the sensing surface with peptides [19]. A metallothionein has been immobilized on gold interfaces to detect Cd^2+^, Zn^2+^, Hg^2+^ and Ni^2+^ [20] and a SAM was also developed for Pt^2+^ using SPR as detector [21].

Very few papers on SPR sensors for Fe^3+^ exist. McIlwee *et al.* investigated the possibility of forming homogeneous, thin chitosan films on the SPR interface to develop a sensor for Fe^3+^ [22]; the problem in this case is the low selectivity due to the well known ability of chitosan to form complexes of similar stability with several heavy metal ions. Moreover the sensor is not suitable for on-line determination. Recently, a polypyrrole thin film sensor based on SPR for detection of Cu(II) and Fe(III) in aqueous solution was proposed [23], however with the same drawbacks highlighted for the previously cited paper.

It is important to underline that in all the studies cited above the SPR sensors are based on a high refractive index prism coated with a thin metallic layer (classical Kretschmann configuration), differently from the presently proposed solution based on POF. POFs are especially advantageous due to their excellent flexibility, easy manipulation, great numerical aperture, large diameter, and the fact that plastic is able to withstand smaller bend radii than glass. The above peculiarities of POFs, that have increased their popularity and competitiveness for telecommunications, are exactly those that are relevant for optical fiber based sensors.

For the detection of Fe(III) DFO is used as ligand for the first time in the case of plasmonic detection, since it is expected to be very selective and sensitive for Fe(III) due to the high stability of the complex. We believe that there is a wide interest in the development of new, simple and selective sensors for metal ions with the innovative SPR detection based on POF.

## 2. Material and Methods

### 2.1. Reagents

All chemicals were of analytical grade. 3-Mercaptapropionic acid (MPA), N-(3-dimethylaminopropyl) -N'-ethylcarbodiimide hydrochloride (EDC), *N*-hydroxysuccinimide (NHS), H_3_PO_4_, NaClO_4_ and NaOH were purchased from Sigma Aldrich (Saint Louis, MO, USA). Deferoxamine mesylate salt (DFO) was obtained from Novartis (Origgio, Italy). All these reagents were used as received. Iron standard solution for ICP of 1,000 mg/L (Fluka, Saint Louis, MO, USA) was used to obtain the proper Fe(III) concentration in the solution phase. Solutions were prepared with ultrapure water (Milli-Q, Merck Millipore, Billerica, MA, USA).

### 2.2. SPR Sensing Platform

The fabricated optical sensor system was realized by removing the cladding of a plastic optical fiber along half the circumference, spin coating on the exposed core a buffer of Microposit S1813 photoresist, and finally sputtering a thin gold film using a sputtering machine [13,14]. The planar gold layer can be employed for depositing a DFO-SAM layer used as specific receptor.

The plastic optical fiber has a PMMA core of 980 μm and a fluorinated polymer cladding of 20 μm. The thickness of the photoresist buffer was about 1.5 μm. The gold film so obtained was 60 nm thick and presented a good adhesion to the substrate, verified by its resistance to rinsing in de-ionized water. The realized sensing region was about 10 mm in length. The refractive indexes of the materials, in the visible range of interest, are about 1.49 for PMMA, 1.41 for fluorinated polymer and 1.61 for Microposit S1813 photoresist.

### 2.3. Preparation of DFO-SAM on the Gold Layer on POF

The procedure is adapted from that reported by Shervedani *et al.* [3] and briefly summarized here. The cleaned gold film on POF was modified by immersion into a 20 mM MPA aqueous solution for 18 h. The formed Au-MPA SAM layer was washed with double-distilled water and then activated for 3 h in 0.1 M phosphate buffer solution (PBS) at pH 5.5, containing 0.002 M EDC and 0.005 M NHS. The activated Au-MPA SAM layer was then rinsed with PBS, dried in N_2_ atmosphere, and used for functionalization. In particular, the activated Au-MPA was immersed in 5 mL of 2.0 mM DFO aqueous solution for 4 h at 25 °C to form Au-MPA-DFO SAM layer on POF. The sensor was removed from the solution and rinsed thoroughly with Milli-Q water to eliminate physically adsorbed species, and dried in N_2_ atmosphere before its use. The sensor is stored in the air and its stability is guaranteed over a period of at least 5 months.

### 2.4. SPR in a POF for Detection of Bio/Chemical Analytes

In the configuration proposed (Figure 1) the surface plasmons are excited by the evanescent wave produced at the interface gold/photoresist layer, when a light beam travels through the plastic optical fiber.

In the optical phenomenon of surface plasmon resonance, a metal-dielectric interface supports a p-polarized electromagnetic wave, namely a Surface Plasmon Wave (SPW), which propagates along the interface. When the light propagates in the POF and the light (only p-polarized) is incident on this metal-dielectric interface in such a way that the propagation constant (and energy) of resultant evanescent wave is equal to that of the SPW, a strong absorption of light takes place as a result of transfer of energy and the output signal exhibits a sharp dip at a particular wavelength known as the resonance wavelength. The observed absorption band is the result of the convolution of different resonance peaks. Each peak is obtained for a specific resonance condition defined by a given angle-wavelength couple.

In SPR sensors with spectral interrogation, the resonance wavelength (*λ*_res_) is determined as a function of the refractive index of the sensing layer (*n*_s_). When artificial receptors are used for bio/chemicals detection, the film on the metal surface selectively recognizes and captures the analyte present in a liquid sample so producing a local increase in the refractive index at the metal surface (*n*_s_). If the refractive index of the sensing layer is altered by *δn*_s_, the resonance wavelength shifts by *δλ*_res_.

The refractive index change Δn_s_ induced by the analyte molecules binding to the recognition elements can be expressed as [24]:

(1) $$\Delta n_{\text{s}}={\left(\frac{dn}{dc}\right)}_{\text{vol}}\Delta c_{\text{s}}$$

where (*dn*/*dc*)_vol_ is the volume refractive index increment, and Δ*c*_s_ is the concentration variation of bound analyte expressed in mass/volume or in any other concentration units in the monolayer. The value of the refractive index increment depends on the structure of the analyte molecules [8,9] and probably also on its interaction with the ligand in the monolayer.

The refractive index increase gives rise to an increase in the propagation constant of Surface Plasmon Wave (SPW) propagating along the metal surface which can be accurately measured, as previously stated.

The sensitivity of an SPR sensor with spectral interrogation can be defined by the shift in resonance wavelength per unit change in refractive index. For a bio-chemical optical sensor with spectral interrogation, the sensitivity is more conveniently defined as:

(2) $$S=\frac{\delta \lambda_{\text{res}}}{\delta c}\left[\frac{nm}{M}\right]$$

In other words, the sensitivity is better defined as the shift in resonance wavelength, Δ*λ*_res_, per unit change in analyte concentration, Δ*c* (nm/M). In the case of a receptors as the DFO-SAM, the monolayer on the gold surface selectively recognizes and captures the analyte present (here Fe(III)) in a liquid sample thus producing a local increase in the refractive index at the metal surface.

### 2.5. Experimental Setup

The experimental setup was arranged to measure the transmitted light spectrum and was characterized by a halogen lamp, illuminating the optical sensor systems (POF of 1,000 μm in diameter), and a spectrum analyzer, as shown in Figure 1.

The employed halogen lamp (Model no. HL-2000-LL, manufactured by Ocean Optics, Dunedin, FL, USA) exhibits a wavelength emission range from 360 nm to 1,700 nm, while the spectrum analyzer detection range was from 200 nm to 850 nm. An Ocean Optics “USB2000+UV-VIS” spectrometer was employed. The spectrometer was finally connected to a computer. The SPR curves along with data values were displayed online on the computer screen and saved with the help of advanced software provided by Ocean Optics.

### 2.6. Procedure

Measurements were performed in 0.5% HNO_3_ (pH = 1.1). Some 20–40 μL of the considered solution were dropped over the gold layer of the sensor, either with or without DFO-SAM, letting the drop to expand over the whole resin block. The transmission spectra were recorded after 5 min incubation. Between successive determinations the sensor was washed by repeatedly—with Milli-Q water and 0.5% HNO_3_. The SPR transmission spectra, normalized to the spectrum achieved with air as the surrounding medium, have been obtained using the Matlab software and the resonance wavelength was extracted for the analytical information.

## 3. Results and Discussion

All the experiments were performed in 0.5% nitric acid solution (pH = 1.1), in order to obviate the precipitation of Fe(III) and to limit its hydrolysis [25]. The complexation of Fe(III) by DFO in solution is strong even at this high acidity [26], as it is in the case of DFO covalently linked to a solid phase (silica) [6]. The fact that Fe(III) is able to interact with the DFO-SAM-POF here considered has been shown by Shervedani *et al.* [3,27] by electrochemical detection. In the present investigation we would like to demonstrate that the association of Fe(III) with the DFO in the monolayer is able to produce an optical signal, due to a refractive index change, despite of the low mass of the substance interacting with the sensor surface (the atomic mass of Fe(III) is only 55.845 amu).

**Figure 2.**
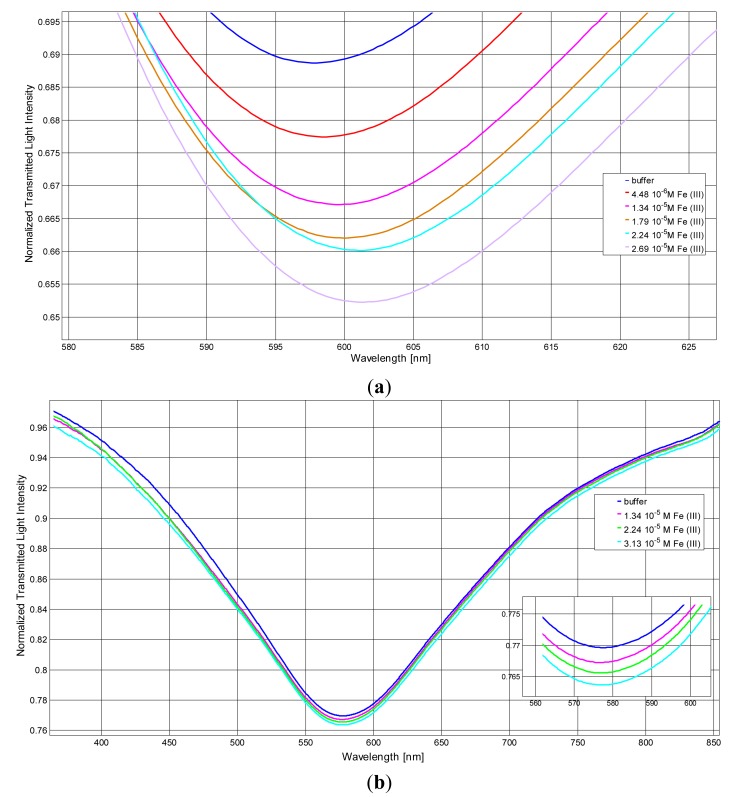
Transmission spectra of DSO-SAM-POF sensor in 0.5% HNO_3_ at increasing concentration of Fe(III). (**a**) measures with DFO-SAM modified gold layer (DFO-SAM sensor); (**b**) measures with bare gold layer (bare sensor). Inset: Zoom of the resonance wavelengths region.

Figure 2a shows the transmission spectra, normalized to the spectrum achieved with air as the surrounding medium, of the sensor in aqueous solution at pH = 1.1 obtained by contacting standard solutions at increasing concentrations of Fe(III).

The resonance wavelength is shifted to higher values by increasing the concentration of Fe(III), which demonstrates that Fe(III) is adsorbed at the derivatized sensor surface, clearly producing an increase of the refraction index of the medium. The spectra of the same solutions at different Fe(III) concentrations, obtained with the optical fiber covered only by gold, without DFO-SAM (bare sensor), are reported for comparison in Figure 2b.

**Figure 3.**
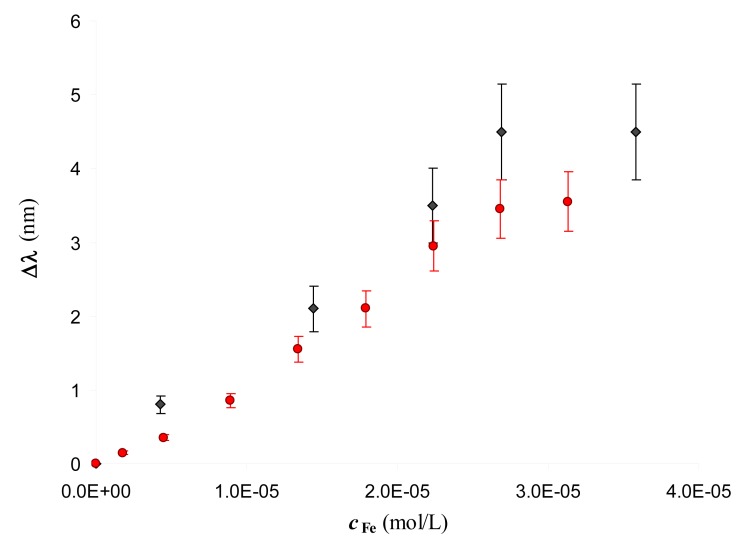
Standardization curves of Fe(III) on two DFO-SAM-POF sensors. Fe(III) standard solutions in 0.5% HNO_3_ (pH = 1.1). 20–40 μl of the standard solution are dropped over the gold layer of the sensor, and the transmission spectra are recorded after 5 min incubation. Experiments performed with two different sensors: DFO-SAM-POF (black diamonds); DFO-SAM-POF (red circles). The error bars correspond to the standard deviation of three replicates for each standard solution.

No wavelength shift at increasing concentrations of Fe(III) is observed at the bare sensor, indicating that the signal, *i.e.*, the resonance wavelength variation, Δλ, is only produced by the interaction of Fe(III) with the functionalized sensor. Notice also that the DFO-SAM sensor exhibits a red-shifted resonance wavelength with respect to that of the bare sensor, which confirms the successful derivatization. The wavelength shift (Δ*λ*) is proportional to the concentration of iron(III) up to about 3 × 10^−5^ M, as reported in Figure 3.

Two experiments, obtained with two different sensors, functionalized following the same procedure, as described in the Experimental part, are reported in order to show the reproducibility. The value of Δ*λ* is similar for the two sensors, even if the resonance wavelengths are poorly reproducible. The response is linear for Fe(III) concentration ranging from 2 × 10^−6^ M to 3 × 10^−5^ M (see below, Equations (6) and (7), for the regression lines associated with the linear part). At higher Fe(III) concentrations a constant value of Δ*λ* is reached, possibly due to the saturation of the available complexing sites of DFO-SAM.

The observed behaviour can be modelled according to the following relationship which is formally similar to the Hill equation, and is widely used for describing site-by-site chemical combinations [16]:

(3) $$\Delta \lambda =S\;c_{\text{DFO}}\frac{K_{c}[\text{Fe}]}{K_{c}[\text{Fe}]+1}$$

Equation (3) has been developed considering Δ*λ* directly proportional to the concentration of the iron(III)-DFO complex in the SAM (*S* indicates the sensitivity). *K_C_* (in M^−1^) is the conditional stability constant of Fe-DFO complex immobilized on the SAM at the considered conditions:

(4) $$K_{C}=\frac{c_{\text{FeDFO}}}{[\text{Fe}]c_{\text{DFO}}}$$

where [Fe] indicates the concentration of iron(III) in the solution phase, *c*_DFO_ the total concentration of DFO not complexed, and *c*_FeDFO_ the concentration of Fe-DFO complex. It is assumed that [Fe] corresponds to the total concentration in the sample (*c*_Fe_), *i.e*., that the amount of iron(III) sorbed is very low compared to the total amount. *K_C_* is a conditional constant at the working pH, since the total concentration of free ligand is considered, not that of the deprotonated species directly involved in the complexation, so by plotting Δ*λ vs*. *c*_Fe_, a straight line with slope *Y c*_DFO_ *K_C_* is expected, while when the iron(III) concentration is much higher than 1/*K_C_*, a line parallel to the abscissa axis with intercept equal to *S c*_DFO_ should be obtained. From the intercept of these two lines, *i.e.*, for [Fe] = 1/*K_C_*, the value of *K_C_* can be evaluated. From the data here obtained, *K_C_* is estimated to be roughly 3 × 10^4^ M^−1^.

To compare the conditional constant *K_C_* in the solid phase with the stability constant in solution, which is usually relative to the following equilibrium [26]:

(5) $$\begin{array}{ll} x\text{Fe}+y\text{H}+z\text{DFO}⇄{\text{Fe}}_{x}{\text{H}}_{y}{\text{DFO}}_{z} & \beta_{\text{xyz}}=\frac{\left[{\text{Fe}}_{x}{\text{H}}_{y}{\text{DFO}}_{z}\right]}{{\left[\text{Fe}\right]}^{x}{\left[\text{H}\right]}^{y}{\left[\text{DFO}\right]}^{z}} \end{array}$$

a simple mathematical transformation has to be done, considering the fraction of the completely deprotonated DFO species (α_DFO_) and the stoichiometric coefficients *x*, *z*, *y*. It has been demonstrated [6] that in the case of DFO immobilized on a silica matrix, the prevailing species for the Fe(III)-DFO combination at the high acidity considered here are FeHDFO or FeH_2_DFO, but not FeDFO. The values of the protonation constants of DFO in solution phase reported by Farkas *et al.* [26] are: log*K*_a1_ = 10.89; log*β*_a2_ = 20.44; log*β*_a3_ = 29.42; log*β*_a4_ = 37.60, from which the molar fraction of the completely deprotonated species of the ligand (*α*_DFO_) at pH = 1.1 is evaluated as log *α*_DFO_ = −29.7. Thus the stability constant of the complexes FeDFO, FeHDFO and FeH_2_DFO, defined according to Equation (5), should be respectively log*β*_110_ = 34.2, log*β*_111_ = 36.2 and log*β*_112_ = 38.2, noticeably different from the values reported for the same complexes in aqueous phase, respectively 30.4, 41.5 and 43.9 [6,26]. The stability constant of the deprotonated complex, calculated from the conditional one, is higher than that in aqueous solution, while the stability constants of the protonated species prevailing at acidic pH, are lower than those in solution phase. These results, although they have to be considered as rough estimates, can be justified considering the repulsive effect of the positive charges at the sensor surface, due to the protonation of the amino groups, on Fe(III).

In view of the application of the considered system as a sensor for Fe(III), it is important to evaluate the dynamic range. In Figure 3 it is seen that the response is linear up to the Fe(III) concentration of about 3 × 10^−5^ M. The equations of the straight line obtained by linear regression, including only points lying on the linear part of the standardization curves, separately for the two experiments reported in Figure 3, are:

(6) $$\begin{array}{lll} \Delta \lambda =1.4(5)\times 10^{5}c_{\text{Fe}}–0.2(1) & {\text{R}}^{2}=0.993 & \left(\text{determination with red circles}\right) \end{array}$$

(7) $$\begin{array}{lll} \Delta \lambda =1.6(8)\times 10^{5}c_{\text{Fe}}–0.01(1) & {\text{R}}^{2}=0.993 & \left(\text{determination with black diamonds}\right) \end{array}$$

The numbers into parentheses are the standard deviations on the parameters.

The detection limit is 2.1 × 10^−6^ M (based on three times the standard deviation of the blank). Lower Fe(III) concentrations were not considered because of the particular care required to process samples containing iron(III) at trace level, easily susceptible to contamination, being iron an ubiquitous element.

### 3.1. Interferences

It is well known that DFO forms much stronger complexes with Fe(III) than with other metal ions [26], even when DFO is fixed in a solid phase [6], so it is expected that this sensor will have good selectivity. A large number of anions and cations have been previously examined by Shervedani *et al.* [27] by an electrochemical sensor based on the same DFO-SAM, for their possible interference in Fe(III) determination. It has been found that the majority of the ions interfere only at very high concentrations.

**Figure 4.**
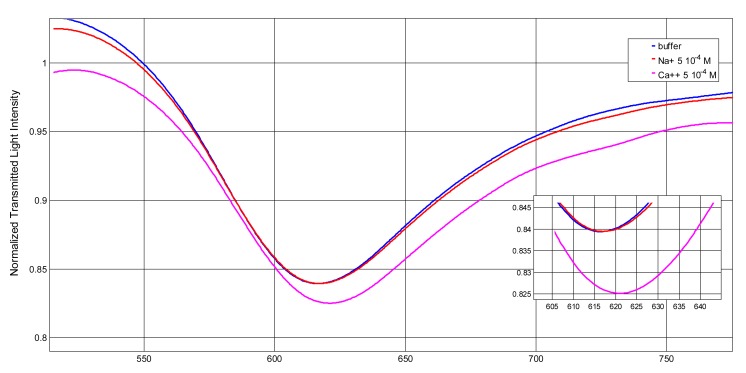
SPR transmission spectra obtained on DFO-SAM-POF sensor for two metal ions: Na^+^ and Ca^2+^. For comparison, the spectrum of 0.5% HNO_3_ (”buffer“ solution) is reported. Inset: Zoom of the resonance wavelengths region.

In the present investigation the effect of Na^+^ concentration on the optical sensor signal is tested in 0.5% HNO_3_, in order to investigate the possible interference of metal ions not complexed by DFO. No variation of the resonance wavelength of the blank solution, even at Na^+^ concentration as high as 0.01 M, occurred. Other metal ions which form complexes by DFO, as reported by Farkas [26], have been examined, as for example copper(II) and calcium(II), even if the conditional constants of the relative complexes with DFO, at the acidity considered in the present study, are very low. Actually no signal variation is produced for concentration of these interferent ions up to 2.5 × 10^−5^ M. Calcium(II) is examined also at higher concentration, *i.e.*, 5 × 10^−4^ M in 0.5% HNO_3_, to simulate real life samples (as for example environmental waters) where it could be present at these concentration levels or higher. The obtained transmission spectra, normalized to the spectrum achieved with air as the surrounding medium, are reported in Figure 4.

**Figure 5.**
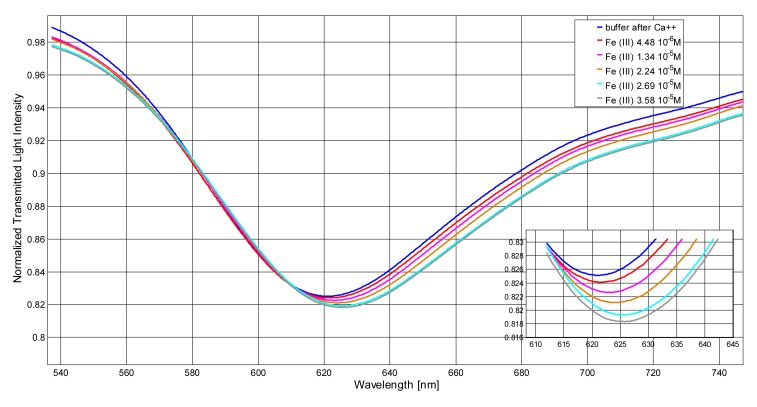
SPR transmission spectra obtained on DFO-SAM-POF sensor for different concentrations of Fe(III). The sensor has been conditioned in Ca^2+^ 5 × 10^−4^ M. Inset: Zoom of the resonance wavelengths region.

Ca(II) at the considered high concentration, is sorbed, since a shift on the resonance wavelength in the same direction of Fe(III) is produced, although the conditional constants of Ca-DFO in solution at high acidity are very low [26]. However, a sensor previously conditioned in Ca^2+^ 5 × 10^−4^ M gave the same sensitivity and dynamic range when used for Fe(III) determination (see Figure 5). It can be concluded that calcium(II), when present at high concentration (higher than 10^−4^M) is sorbed on DFO-SAM-POF producing a different base signal, which however has no influence on the sensitivity of the iron(III) determination. This could tentatively be ascribed to the presence of different complexation sites in SAM for Ca(II) and Fe(III) at least in acidic solutions.

### 3.2. Multiple Use of the Sensor

A rapid washing of the sensor with 20 μL 0.5% HNO_3_, contacted for 5 min, does not produce any signal variation, both in the case of iron(III) and calcium(II). This could be ascribed to very slow kinetics for the release of these metal ions from DFO-SAM.

**Figure 6.**
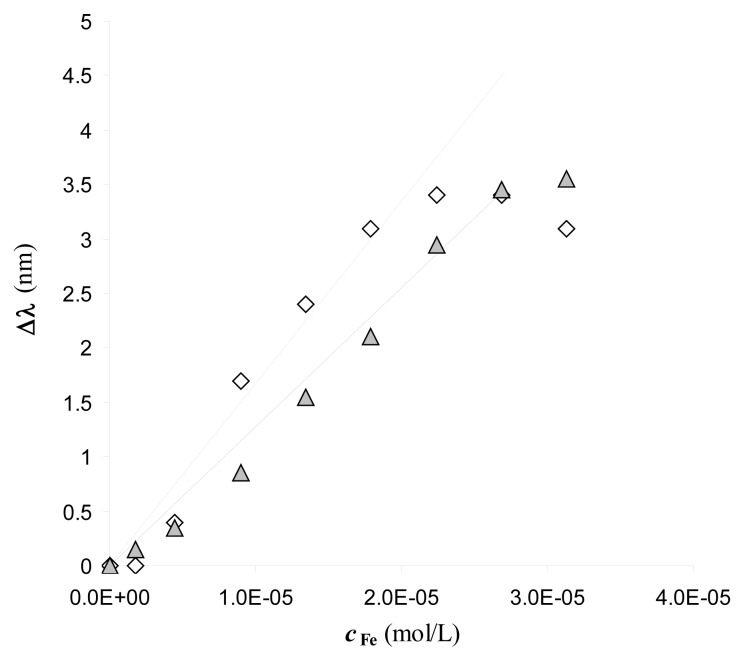
Standardization curves of Fe(III) on DFO-SAM-POF sensor. 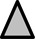: Δ*λ* obtained from the transmission spectrum in 0.5% M HNO_3_ at increasing Fe(III) concentration; ♢: Δ*λ* obtained from the transmission spectrum in 0.01 M Na_2_EDTA (washing solution) after each determination.

According to a previously suggested method [28], applied to a different immobilized DFO system, washings have been performed with 20 μL of 0.01 M Na_2_EDTA solution at the natural pH (about 4.7) for 5 min. This acidity for the washing solution was selected in order to increase the conditional complexation constant for the metal-EDTA complex. Unexpectedly, the analysis in a fresh Na_2_EDTA 0.01 M solution after the washing, showed an increase of the resonance wavelength instead of a decrease. This behaviour is tentatively ascribed to the variation of the refractive index of DFO-SAM phase due to the deprotonation of DFO at higher pH, 4.7. A combined effect of a resonance wavelength increase, due to the deprotonation of the ligand, and a resonance wavelength decrease due to the ion exchange of Fe(III) with Na^+^ ions in the SAM phase, could be hypothesized. This seems not to be the case since the signal recorded in Na_2_EDTA washing solution is dependent on the Fe(III) concentration in the sample contacted immediately before washing. This is seen in Figure 6, where the curve obtained by recording the spectra in 20 μL of 0.01 M Na_2_EDTA immediately after the contact with the sample solution, is compared with the usual calibration curve obtained by measuring Δ*λ* in the Fe(III) sample solutions.

Other elution methods have been tested, as for example electrochemical reduction of Fe(III), but with no success. Despite of the apparent irreversibility of the Fe(III) sorption on the DFO-SAM-POF sensor surface, it has to be noticed that a freshly prepared sensor can be used for a whole standard calibration or for Fe(III) determination in an unknown sample by the standard addition method, maintaining a good linearity until the saturation is reached, thanks to the fact that the free DFO concentration in the SAM phase can be considered as constant up to an external solution Fe(III) concentration of 3 × 10^−5^ M, as reported above.

## 4. Conclusions

A sensor based on SPR in a POF has been developed for the interesting metal ion Fe(III). It relies on a strong ligand, DFO, which has been covalently linked to a gold surface as a self-assembled monolayer, according to a previously reported method [3,27]. A useful optical response, ascribable to the Fe(III)-DFO interaction on the solid surface of the sensor, has been obtained, despite the low mass of the considered metal ion.

The detection limit is relatively high, compared with that claimed by applying other transduction methods [27] and with the concentration levels actually found in real life samples. Evidently the conditional constant for the combination of Fe(III) with DFO in the SAM at relatively acidic pH is not high enough to make possible the combination of a sufficient amount of Fe(III) to give an optical signal (resonance wavelength shift) significantly different from the noise, when the Fe(III) concentration is lower than 2 × 10^−6^ M. Work is in progress to improve the sensitivity of the method, for example by modifying the geometry of the optical fiber, as suggested in a previous investigation [16]. While some interferences from other metal ions at high concentration, in particular from calcium(II) ions, have been detected, they seem to influence only the base signal, and not the sensitivity and the linearity range. More important seems to be the solution acidity effect, and this aspect too is under investigation. It is important to underline that this work presents just the proof of principle of the new optical fiber sensor based on DFO-SAM receptor. The reported experimental results open the door to a new approach in the chemical optical sensor topic, toward remote, small size, selective and low cost sensor systems for Fe(III) detection. This kind of bio-chemical sensors is good for application as disposable sensors because of the easy preparation, the high stability and the very low cost.
